# Supplementary material for: Bacterial communities in the digester bed and liquid effluent of a microflush composting toilet system
Source: PeerJ. 2018 Dec 6;6:e6077. doi: 10.7717/peerj.6077 (PMC6286801; doi:10.7717/peerj.6077)
Supplement: Supplemental Information 1 — * based on certificate of analysis. [file peerj-06-6077-s001.docx]

Supplementary Table 1. Expected species and concentrations in mock community compared to OTU classifications and sequence counts

| Expected | Concentration* | OTU: Classification | Sequences |
| --- | --- | --- | --- |
| *Acinetobacter baumannii* | 82 pg/µl | Otu00050: *Acinetobacter* | 953 |
| *Actinomyces odontolyticus* | 10 pg/µl | Otu00812: *Actinomyces* | 20 |
| *Bacillus cereus* | 450 pg/µl | Otu00046: Bacillales | 5813 |
| *Bacteroides vulgatus* | 7.6 pg/µl | Otu00040: *Bacteroides* | 130 |
| *Clostridium beijerinckii* | 440 pg/µl | Otu00041: *Clostridium* | 6747 |
| *Deinococcus radiodurans* | 10 pg/µl | Otu00781: *Deinococcus* | 72 |
| *Enterococcus faecalis* | 7.0 pg/µl | Otu00686: Lactobacillales | 26 |
| *Escherichia coli* | 6800 pg/µl | Otu00007 and Otu01306: *Escherichia/Shigella* | 33493 |
| *Helicobacter pylori* | 86 pg/µl | Otu00151: *Helicobacter* | 1131 |
| *Lactobacillus gasseri* | 32 pg/µl | Otu00271: *Lactobacillus* | 481 |
| *Listeria monocytogenes* | 50 pg/µl | Otu00332: *Listeria* | 357 |
| *Neisseria meningitidis* | 58 pg/µl | Otu00170: *Neisseria* | 998 |
| *Propionibacterium acnes* | 88 pg/µl | Otu00987: *Propionibacterium* | 43 |
| *Pseudomonas aeruginosa* | 1600 pg/µl | Otu00039: *Pseudomonas* | 2274 |
| *Rhodobacter sphaeroides* | 14000 pg/µl | Otu00001 and Otu01750: Rhodobacteraceae | 21176 |
| *Staphylococcus aureus* | 590 pg/µl | Otu00002: *Staphylococcus* | 57086 |
| *Staphylococcus epidermidis* | 5100 pg/µl |  |  |
| *Streptococcus agalactiae* | 32 pg/µl | Otu00004, Otu00067, Otu01175 and Otu00662: *Streptococcus* | 50075 |
| *Streptococcus mutans* | 4100 pg/µl |  |  |
| *Streptococcus pneumoniae* | 5.5 pg/µl |  |  |

* based on certificate of analysis
