# Supplementary figures and images for: Bacterial communities in the digester bed and liquid effluent of a microflush composting toilet system
Source: PeerJ. 2018 Dec 6;6:e6077. doi: 10.7717/peerj.6077 (PMC6286801; doi:10.7717/peerj.6077)

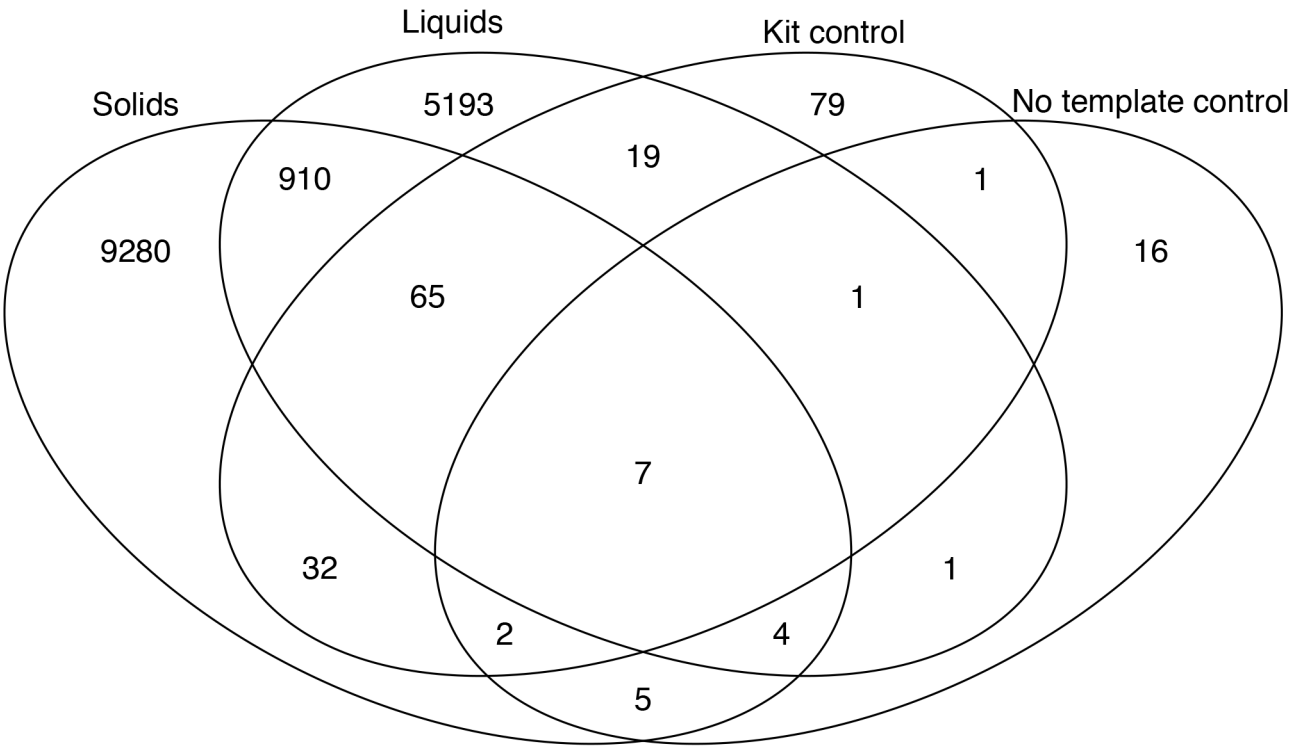

Supplement: Supplemental Information 2 — Distribution of unique sequences in filter-digester bed samples (solids), liquid effluent samples (liquids), control to test for bacterial DNA contamination of genomic DNA extraction materials (kit control) and control to test for bacterial DNA contamination of PCR materials (no template control). [file peerj-06-6077-s002.pdf]

**A**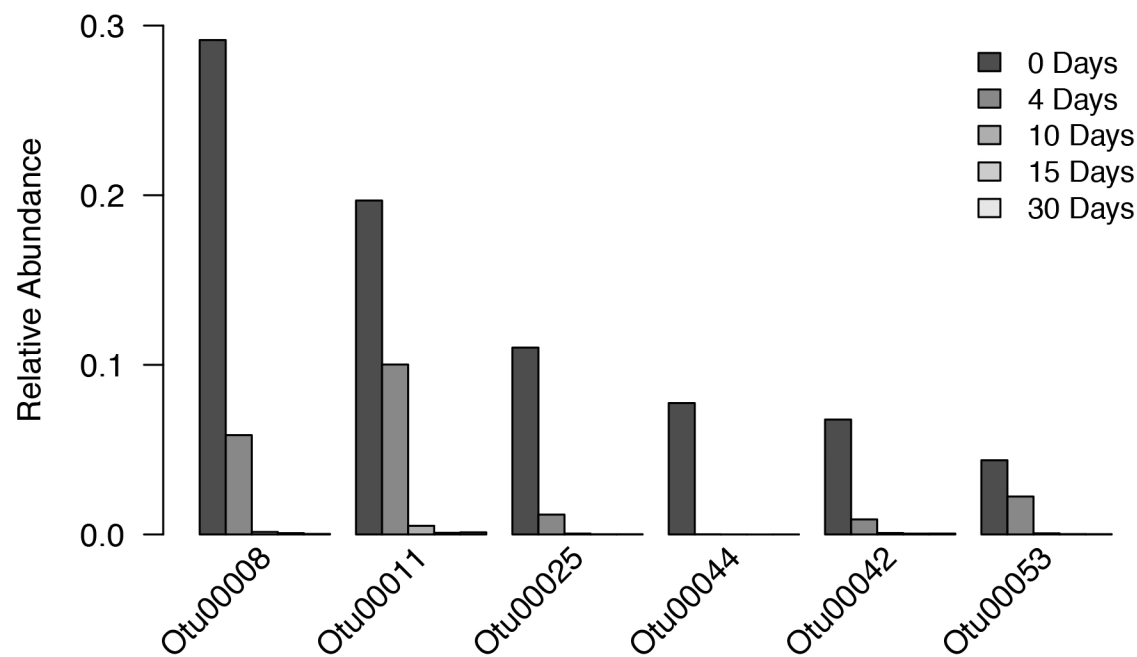**B**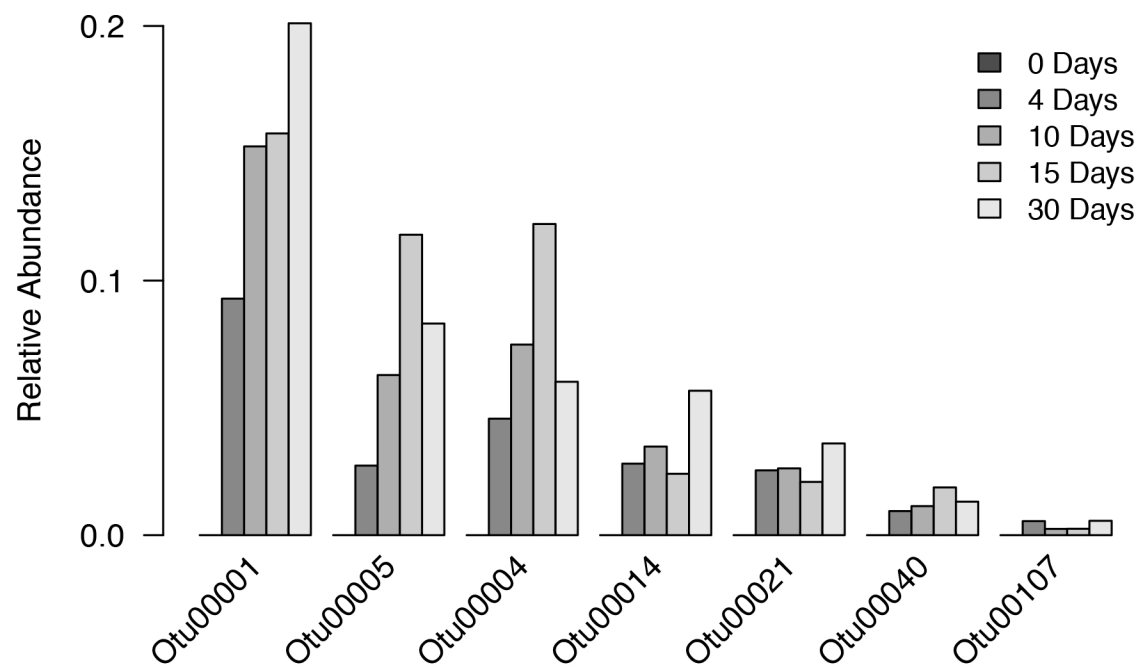

Supplement: Supplemental Information 3 — A. Unclassified OTUs occurring at a relative abundance >2% in the time zero sample, shown for all filter-digester bed samples. B. Unclassified OTUs occurring at a mean relative abundance >2% in the four subsequent samples (day 4–day 30), shown for all filter-digester bed samples. [file peerj-06-6077-s003.pdf]

**A**

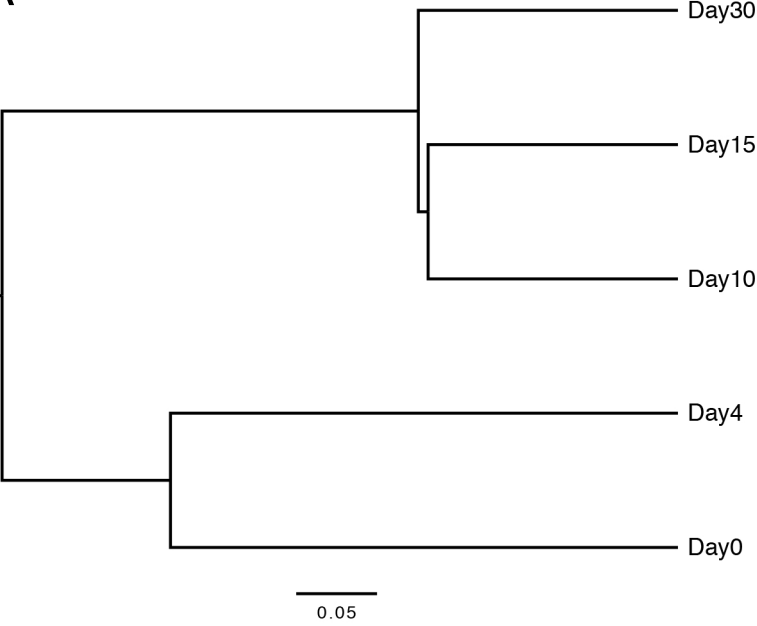

**B**

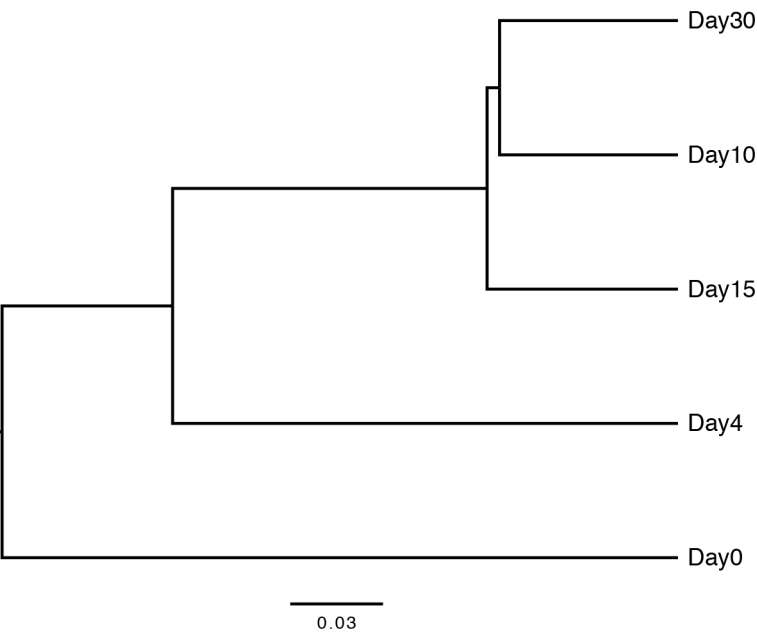

Supplement: Supplemental Information 4 — A. Dendrogram constructed with UPGMA from a distance matrix calculated using Bray-Curtis dissimilarity. B. Dendrogram constructed with UPGMA from a distance matrix calculated using weighted UniFrac distance. [file peerj-06-6077-s004.pdf]

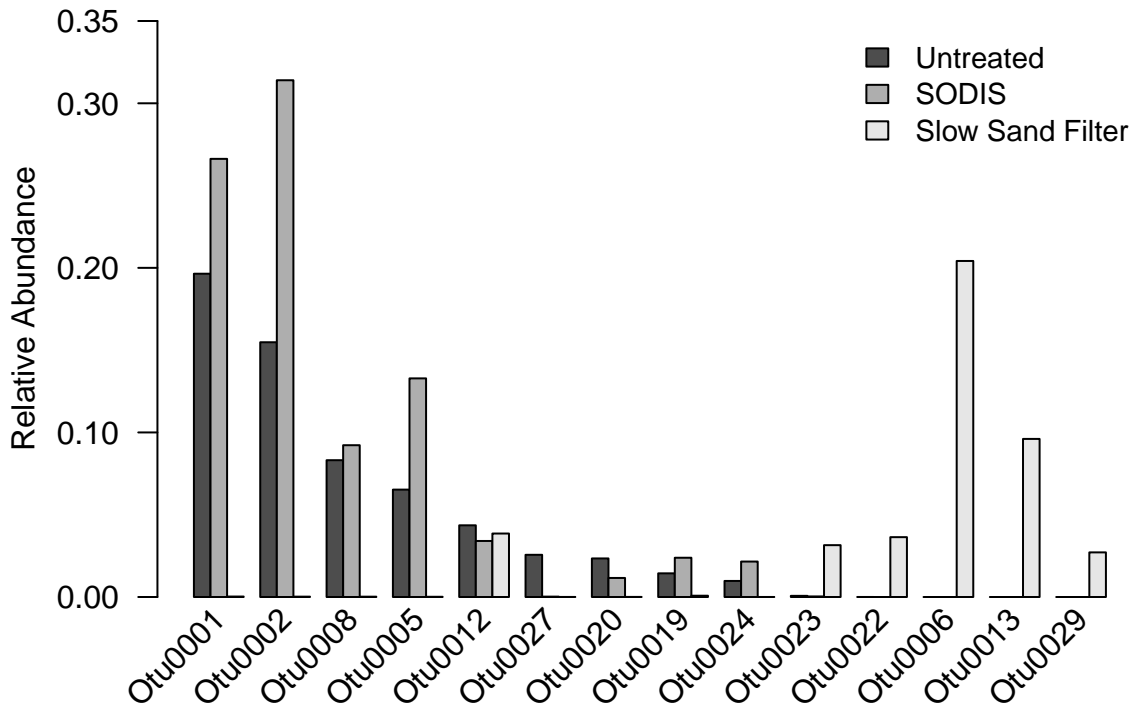

Supplement: Supplemental Information 5 [file peerj-06-6077-s005.pdf]
